# Physiological, hyaluronan-selected intracytoplasmic sperm injection for infertility treatment (HABSelect): a parallel, two-group, randomised trial

**DOI:** 10.1016/S0140-6736(18)32989-1

**Published:** 2019-02-02

**Authors:** David Miller, Susan Pavitt, Vinay Sharma, Gordon Forbes, Richard Hooper, Siladitya Bhattacharya, Jackson Kirkman-Brown, Arri Coomarasamy, Sheena Lewis, Rachel Cutting, Daniel Brison, Allan Pacey, Robert West, Kate Brian, Darren Griffin, Yakoub Khalaf

**Affiliations:** a Leeds Institute of Cardiovascular and Metabolic Medicine, University of Leeds Laboratories, University of Leeds, Leeds, UK; b Dental Translational and Clinical Research Unit, Leeds National Institute for Health Research Clinical Research Facility, University of Leeds, Leeds, UK; c Leeds Institute of Health Sciences, University of Leeds, Leeds, UK; d The Leeds Centre for Reproductive Medicine, Seacroft Hospital, Leeds, UK; e Pragmatic Clinical Trials Unit, Centre for Primary Care and Public Health, Queen Mary University of London, London, UK; f School of Medicine, College of Biomedical and Life Sciences, Cardiff University, Cardiff, UK; g Institute of Metabolism and Systems Research, College of Medical & Dental Sciences, University of Birmingham, Birmingham, UK; h Birmingham Women's Hospital, Birmingham Women's and Children's National Health Service (NHS) Foundation Trust, Birmingham, UK; i Examen Ltd, Belfast, UK; j Sheffield Teaching Hospitals NHS Trust, Sheffield, UK; k Department of Reproductive Medicine, Manchester University Hospitals NHS Foundation Trust, Manchester Academic Health Sciences Centre, St Mary's Hospital, Manchester, UK; l Department of Oncology & Metabolism, University of Sheffield, Sheffield, UK; m Royal College of Obstetricians and Gynaecologists, London, UK; n School of Biosciences, University of Kent, Canterbury, UK; o Guy's and St Thomas' NHS Foundation Trust, Guy's Hospital, London, UK

## Abstract

**Background:**

Sperm selection strategies aimed at improving success rates of intracytoplasmic sperm injection (ICSI) include binding to hyaluronic acid (herein termed hyaluronan). Hyaluronan-selected sperm have reduced levels of DNA damage and aneuploidy. Use of hyaluronan-based sperm selection for ICSI (so-called physiological ICSI [PICSI]) is reported to reduce the proportion of pregnancies that end in miscarriage. However, the effect of PICSI on livebirth rates is uncertain. We aimed to investigate the efficacy of PICSI versus standard ICSI for improving livebirth rates among couples undergoing fertility treatment.

**Methods:**

This parallel, two-group, randomised trial included couples undergoing an ICSI procedure with fresh embryo transfer at 16 assisted conception units in the UK. Eligible women (aged 18–43 years) had a body-mass index of 19–35 kg/m^2^ and a follicle-stimulating hormone (FSH) concentration of 3·0–20·0 mIU/mL or, if no FSH measurement was available, an anti-müllerian hormone concentration of at least 1·5 pmol/L. Eligible men (aged 18–55 years) had not had a vasovasostomy or been treated for cancer in the 24 months before recruitment and were able, after at least 3 days of sexual abstinence, to produce freshly ejaculated sperm for the treatment cycle. Couples were randomly assigned (1:1) with an online system to receive either PICSI or a standard ICSI procedure. The primary outcome was full-term (≥37 weeks' gestational age) livebirth, which was assessed in all eligible couples who completed follow-up. This trial is registered, number ISRCTN99214271.

**Findings:**

Between Feb 1, 2014, and Aug 31, 2016, 2772 couples were randomly assigned to receive PICSI (n=1387) or ICSI (n=1385), of whom 2752 (1381 in the PICSI group and 1371 in the ICSI group) were included in the primary analysis. The term livebirth rate did not differ significantly between PICSI (27·4% [379/1381]) and ICSI (25·2% [346/1371]) groups (odds ratio 1·12, 95% CI 0·95–1·34; p=0·18). There were 56 serious adverse events in total, including 31 in the PICSI group and 25 in the ICSI group; most were congenital abnormalities and none were attributed to treatment.

**Interpretation:**

Compared with ICSI, PICSI does not significantly improve term livebirth rates. The wider use of PICSI, therefore, is not recommended at present.

**Funding:**

National Institute for Health Research Efficacy and Mechanism Evaluation Programme.

## Introduction

Globally, between 2008 and 2010, more than 4·7 million treatment cycles of assisted reproduction techniques were performed, of which around half involved intracytoplasmic sperm injection (ICSI),^1^ leading to the birth of 1·14 million babies.^2^ ICSI, which was originally developed to treat male infertility,^3^ is normally used to treat men with few or abnormal sperm and is associated with livebirth rates of around 24% per treatment cycle, a frequency that has remained unchanged in the past decade.^1^, ^2^ ICSI is becoming the preferred treatment for infertility, although it might not confer any substantial advantage over in-vitro fertilisation (IVF) in cases of non-male-factor infertility.^1^ Additionally, unlike IVF, ICSI does not help prevent dysfunctional sperm from entering the egg.^4^ Screening sperm on the basis of quality before ICSI is one possible solution to this problem,^5^ potentially assisting embryologists in selecting the best sperm for injection and accordingly improving treatment success rates.

Hyaluronic acid (herein termed hyaluronan) is a biologically active molecule that is also a major component of the extracellular matrix surrounding the oocyte-cumulus complex.^6^ Several small clinical studies,^7^, ^8^, ^9^, ^10^, ^11^ including three randomised trials,^7^, ^8^, ^9^ reported that ICSI with hyaluronan-selected sperm (so-called physiological ICSI [PICSI]) improved embryo quality and livebirth rates and decreased miscarriage rates compared with ICSI with sperm selected using standard methods. In these studies, couples who benefited most from treatment had low hyaluronan–sperm binding scores, and in one study,^11^ baseline hyaluronan–sperm binding scores were used to decide who should be treated with PICSI.

**Research in context**

**Evidence before this study**

Although the advent of intracytoplasmic sperm injection (ICSI) has transformed the treatment of male infertility, success rates in terms of livebirths per treated couple have remained reasonably static in the past 10 years at around 25%. Demand for fertility treatment around the world is rising, and more than half of all treatment cycles now involve ICSI. Research aimed at increasing livebirth rates has focused mainly on women, but an increasing interest in male fertility has led to the development of sperm selection techniques to allow embryologists to select, with confidence, the best sperm for ICSI and potentially improve success rates. The naturally occurring organic polymer, hyaluronic acid (herein termed hyaluronan), is thought to select sperm with good DNA integrity and low rates of aneuploidy. Hyaluronan-based selection of sperm (so-called physiological ICSI [PICSI]) has been reported to increase livebirth rates and decrease miscarriage rates, and a multicentre randomised controlled trial of PICSI efficacy, although considerably larger than previous trials, did not report livebirth rates. Furthermore, the importance of the hyaluronan–sperm binding score in determining the likely benefit of sperm selection to the couple remains uncertain.

**Added value of this study**

To our knowledge, HABSelect is the largest randomised trial of PICSI to date and the first to provide a robust measure of livebirth. PICSI did not significantly increase the term livebirth rate compared with standard ICSI, but a significant decrease was observed in miscarriage rates among couples in the PICSI group. There were no differences between groups in any other outcome, and the hyaluronan–sperm binding score did not seem to predict or affect the outcomes of treatment.

**Implications of all the available evidence**

All of the available evidence suggests that hyaluronan-based sperm selection decreases miscarriage rates after ICSI, but not sufficiently to affect livebirth rates. The mechanism through which PICSI might reduce rates of miscarriage is being investigated in an ongoing independent analysis.

We aimed to test the hypothesis that PICSI improves full-term (≥37 weeks' gestational age) livebirth rates compared with ICSI.

## Methods

### Study design and participants

HABSelect was a parallel, two-group, randomised trial at 16 assisted conception units licensed by the Human Fertilisation and Embryology Authority (HFEA) in the UK.^12^ Eligible couples were undergoing an ICSI procedure with fresh embryo transfer. Eligible women were aged 18–43 years; had a body-mass index (BMI) between 19 kg/m^2^ and 35 kg/m^2^; and had a follicle-stimulating hormone (FSH) concentration between 3·0 mIU/mL and 20·0 mIU/mL or, if no FSH measurement was available, an anti-müllerian hormone (AMH) concentration of at least 1·5 pmol/L. Eligible men were aged 18–55 years; had not had a vasovasostomy or been treated for cancer in the 24 months before recruitment; and were able, after at least 3 days of sexual abstinence, to produce freshly ejaculated sperm for the treatment cycle. Couples were excluded if they were using donor or frozen gametes or undergoing split IVF–ICSI.

This study was approved by the National Research Ethics Service (approval number 13/YH/0162). Final approval for participation in the trial was obtained from the doctor (or team) in charge of treatment. Written informed consent was obtained from all participants.

### Randomisation and masking

Embryologists at research sites randomly assigned (1:1) couples, using an online randomisation system, to undergo PICSI or standard ICSI. Randomisation was done by minimisation with a random component, stratified by site, to balance for maternal age (<35 years *vs* ≥35 years), paternal age (<35 years *vs* ≥35 years), previous miscarriages (none *vs* one or two *vs* more than two), and concentrations of hormonal indicators of ovarian reserve (FSH <6·0 mIU/mL *vs* ≥6·0 mIU/mL; AMH <17·0 pmol/L *vs* ≥17·0 pmol/L). The randomisation system revealed allocations only after treatment assignment was complete. Trial participants, research staff collecting outcome data, and all trial team members who contributed to the statistical analysis plan were masked to treatment allocation until the database was locked and the statistical analysis plan was signed off. It was impractical to conceal allocation from the embryologists performing the intervention.

### Procedures

Women at each treatment centre underwent ovarian stimulation with long-agonist, short-agonist, or antagonist regimens according to local protocols.^12^ Similarly, egg retrieval, laboratory culture, and ICSI followed locally approved protocols.^12^ PICSI plates were obtained from Origio (Reigate, UK), and sperm selection was done according to the supplier's instructions and only after local training in the procedure. Treatments concluded with the transfer of one to three fresh embryos on days 3 or 5 after fertilisation, which were selected on the basis of their morphology.

We calculated hyaluronan–sperm binding scores on the day of treatment for prepared, washed semen using the Hydak slide (Origio, Reigate, UK), according to the supplier's instructions. These scores were calculated as the number of immobilised sperm divided by the total number of sperm, multiplied by 100. To aid comparisons with other studies that calculated hyaluronan–sperm binding scores,^8^, ^11^, ^13^ we stratified the scores into those that were 65% or less and those greater than 65%. Follow-up data were collected according to approved, trial-specific case report forms^12^ and from routine patient records.

### Outcomes

The primary outcome was full-term (≥37 weeks' gestational age) livebirth. Secondary outcomes were clinical pregnancy (defined as the presence of a fetal heartbeat or gestational sac at 6–9 weeks after fresh embryo transfer), miscarriage (defined as pregnancy loss after confirmation of clinical pregnancy), and livebirth before 37 weeks' gestational age (henceforth described as premature birth).

### Statistical analysis

On the basis of HFEA longitudinal data, we estimated that the term livebirth rate in the ICSI group would be about 24%. To detect a 5% increase (from 24% to 29%) in the PICSI group, with 90% power at the 5% significance level, 3266 couples were required. We aimed to recruit 3700 couples to allow for a 10% dropout rate. However, because of poorer than expected recruitment, in October, 2015, the trial steering committee (who were masked to treatment assignment) recommended that the target for power should be revised to 80%, meaning that 2444 couples were required for the primary analysis.

Primary and secondary endpoints were assessed in the modified intention-to-treat population, which included all couples who completed follow-up, analysed in the groups to which they were randomly assigned. Our statistical analysis plan prespecified that if more than 5% of primary outcome data were missing, we would do sensitivity analyses to investigate the effects of departures from the missing-at-random assumption on our conclusions. Primary analysis of primary and secondary outcomes was done using a mixed effects logistic regression model adjusted for the minimisation factors (maternal age, paternal age, number of previous miscarriages, and hormonal indicators of ovarian reserve) and a random effect for site. We used cubic splines with three knots (knot locations based on Harrell's recommendations) to adjust for maternal and paternal ages.^14^, ^15^ A prespecified sensitivity analysis of the primary outcome was also done, with adjustment for additional covariates, including mother's BMI, ethnicity, history of pregnancy, smoking status, and hormonal treatment received (long agonist, short agonist, or antagonist).

Effects of treatment are presented as odds ratios (ORs) with 95% CIs. Absolute risk differences with 95% CIs were calculated from unadjusted logistic regression models with the delta method.^16^

We did prespecified subgroup analyses to investigate whether the effect of treatment on the primary outcome was modified by hyaluronan–sperm binding score, maternal age, previous miscarriage, or hormonal indicators of ovarian reserve. Following database locking, unmasking, and analysis, we were alerted to an unexpected and significant difference in miscarriage rates between trial groups. Therefore, we did a post-hoc subgroup analysis to investigate whether hyaluronan–sperm binding score, maternal age, previous miscarriage, or hormonal indicators of ovarian reserve modified the effect of treatment on miscarriage rates. All analyses were done with Stata version 14. An α value of 5% or less was considered significant. The full statistical analysis plan is available in the appendix.

### Role of the funding source

The funder of the study had no role in study design, data collection, data analysis, data interpretation, or writing of the report. The corresponding author and statistical support authors (GF and RH) had full access to all the data in the study, and the corresponding author had final responsibility for the decision to submit for publication.

## Results

Between Feb 1, 2014, and Aug 31, 2016, 2772 couples were randomly assigned to receive PICSI (n=1387) or standard ICSI (n=1385; figure). Six couples who did not meet eligibility criteria were excluded after randomisation, and 14 couples were lost to follow-up; thus, 2752 couples were included in the primary analysis (figure).

**Figure:**
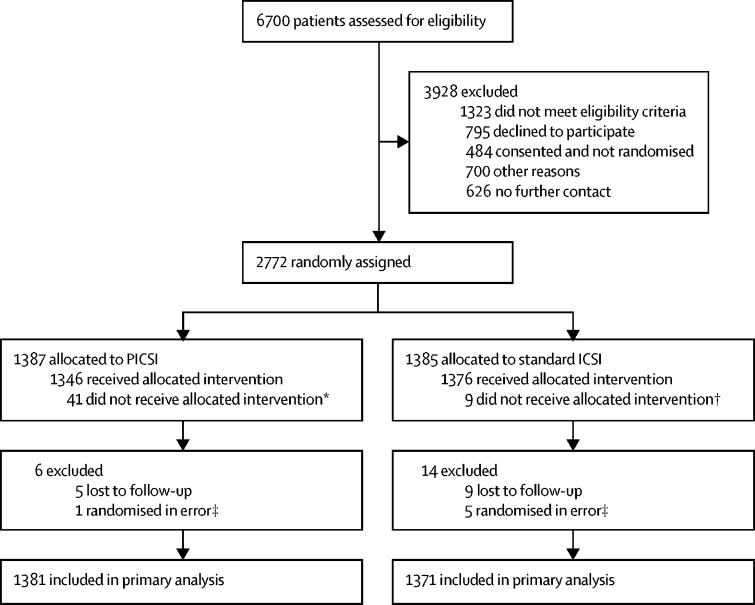
Trial profile PICSI=physiological intracytoplasmic sperm injection. ICSI=intracytoplasmic sperm injection. IVF=in-vitro fertilisation. *Three received IVF, two received IVF–ICSI split cycle, and 36 received ICSI. †Five received IVF, three received IVF–ICSI split cycle, and one received PICSI. ‡These individuals were found to not meet eligibility criteria after randomisation.

Baseline and treatment characteristics were well balanced between groups (Table 1, Table 2). The median sperm concentration before treatment (11·0 × 10^6^ per mL [IQR 3·5–30·0]; table 1) was lower than the WHO lower reference value (5th centile: 15·0 × 10^6^ per mL)^17^ but had risen to this concentration by the day of treatment (table 2).

**Table 1:** Baseline characteristics

		**PICSI (n=1386)**	**ICSI (n=1380)**
**Male partner**			
Age (years)		36·1 (5·5; n=1386)	35·9 (5·4; n=1380)
	≥35 years	812/1386 (59%)	803/1380 (58%)
BMI (kg/m^2^)		27·3 (4·6; n=570)	27·0 (4·2; n=549)
Ethnicity			
	White	1047/1386 (76%)	1078/1380 (78%)
	Asian	193/1386 (14%)	166/1380 (12%)
	Black	49/1386 (4%)	45/1380 (3%)
	Other	36/1386 (3%)	45/1380 (3%)
	Not stated	61/1386 (4%)	46/1380 (3%)
Current smoker		68/1365 (5%)	65/1353 (5%)
	Cigarettes per day	8·0 (5·5; n=63)	8·5 (5·2; n=59)
Consumes alcohol		771/1304 (59%)	791/1300 (61%)
	Units per week	7·7 (6·3; n=724)	7·7 (6·8; n=740)
Recreational drug use		7/1303 (1%)	6/1286 (<1%)
Sperm concentration (×10^6^ per mL)*		11·0 (3·5–29·5; n=1335)	11·0 (3·6–31·0; n=1338)
ICSI recommended based on semen assessment		1268/1320 (96%)	1245/1310 (95%)
**Female partner**			
Age (years)		33·6 (4·4; n=1386)	33·7 (4·3; n=1380)
	≥35 years	618/1386 (45%)	617/1380 (45%)
BMI (kg/m^2^)		24·7 (3·5; n=1368)	24·4 (3·5; n=1360)
Ethnicity			
	White	1029/1386 (74%)	1049/1380 (76%)
	Asian	214/1386 (15%)	189/1380 (14%)
	Black	45/1386 (3%)	46/1380 (3%)
	Other	52/1386 (4%)	55/1380 (4%)
	Not stated	46/1386 (3%)	41/1380 (3%)
Current smoker		31/1375 (2%)	20/1368 (1%)
	Cigarettes per day	6·4 (3·3; n=28)	6·3 (3·6; n=20)
Consumes alcohol		646/1340 (48%)	673/1328 (51%)
	Units per week	5·1 (4·3; n=614)	5·1 (4·7; n=634)
Recreational drug use		1/1317 (<1%)	1/1302 (<1%)

Data are mean (SD) or n/N (%), unless otherwise indicated. n refers to the number of patients for whom data were available. PICSI=physiological intracytoplasmic sperm injection. ICSI=intracytoplasmic sperm injection. BMI=body-mass index.

* Data are median (IQR).

**Table 2:** Treatment characteristics

		**PICSI (n=1386)**	**ICSI (n=1380)**
**Semen pre-preparation assessment**			
Semen volume (mL)		2·9 (1·4;n=1338)	3·0 (1·5;n=1332)
Sperm concentration (×10^6^ per mL)*		14·7 (4·0–35·0;n=1236)	16·0 (5·0–36·4;n=1223)
Forward progressive motility (%)		39·5 (20·1;n=1216)	40·8 (20·3;n=1198)
**Semen post-preparation assessment**			
Sperm processing method			
	Swim-up	18/1343 (1%)	19/1337 (1%)
	Density gradient centrifugation	1044/1343 (78%)	1028/1337 (77%)
	Direct centrifugation	191/1343 (14%)	198/1337 (15%)
	Other	89/1343 (7%)	90/1337 (7%)
	Not processed	1/1343 (<1%)	2/1337 (<1%)
Forward progressive motility (%)		68·6 (28·1;n=1161)	69·5 (27·5;n=1140)
**Hyaluronan–sperm binding score**			
≤25%		86/963 (9%)	74/947 (8%)
26–65%		188/963 (20%)	181/947 (19%)
>65%		689/963 (72%)	692/947 (73%)
**Oocyte collection**			
Number of eggs collected per couple		10·9 (6·3;n=1345)	10·8 (6·3;n=1337)
Number of metaphase II oocytes injected with sperm		8·7 (5·1; n=1341)	8·5 (5·1; n=1331)

Data are mean (SD) or n/N (%), unless otherwise stated. n refers to the number of patients for whom data were available. PICSI=physiological intracytoplasmic sperm injection. ICSI=intracytoplasmic sperm injection.

* Data are median (IQR).

The term livebirth rate was 27·4% in the PICSI group and 25·2% in the standard ICSI group (OR 1·12, 95% CI 0·95–1·34; p=0·18; table 3; appendix). Similar results were obtained in the prespecified sensitivity analysis of the primary outcome (table 3). We did not find evidence of differential effects of treatment on the primary outcome according to hyaluronan–sperm binding scores, maternal age, previous miscarriage, maternal FSH concentrations, or paternal sperm concentrations (appendix).

**Table 3:** Trial outcomes

		**PICSI**	**ICSI**	**Absolute difference (95% CI)**	**Odds ratio (95% CI)**	**p value**
**Term livebirth**						
	Primary analysis*	27·4% (379/1381)	25·2% (346/1371)	2·2% (−1·1 to 5·5)	1·12 (0·95 to 1·34)	0·18
	Sensitivity analysis†	27·5% (379/1379)	25·3% (346/1370)	2·2% (−1·1 to 5·5)	1·13 (0·95 to 1·34)	0·17
**Secondary endpoints**						
Clinical pregnancy		35·2% (487/1382)	35·7% (491/1375)	−0·5% (−4·0 to 3·1)	0·98 (0·84 to 1·15)	0·80
Miscarriage		4·3% (60/1381)	7·0% (96/1371)	−2·7% (−4·4 to −0·9)	0·61 (0·43 to 0·84)	0·003
Premature birth		3·3% (46/1381)	3·3% (45/1371)	0·0% (−1·3 to 1·4)	1·02 (0·67 to 1·55)	0·94
**Exploratory endpoints**						
Fertilisation rate (%)‡		66% (24·0)	69% (24·0)	3·0% (−0·47 to 6·5)	1·15 (0·98 to 1·34)	0·09
Biochemical pregnancy		39·5% (546/1383)	39·5% (544/1377)	0·0% (−4·0 to 4·0)	1·00 (0·86 to 1·17)	0·99

Data are % (n/N), unless otherwise stated. PICSI=physiological intracytoplasmic sperm injection. ICSI=intracytoplasmic sperm injection.

* Adjusted for maternal age, previous miscarriage, and hormonal indicators of ovarian reserve.

† Adjusted for hyaluronan–sperm binding score, maternal age, previous miscarriage, and hormonal indicators of ovarian reserve. Odds ratios are shown alongside absolute differences.

‡ Data are mean (SD); denominators were 1386 for the PICSI group and 1380 for the ICSI group.

The proportions of couples with clinical pregnancy or premature birth were not significantly different between groups (table 3). By contrast, the proportion of couples whose clinical pregnancy ended in miscarriage was significantly lower in the PICSI group than in the ICSI group (table 3; appendix). We found no evidence of differential effects of treatment on miscarriage rates according to hyaluronan–sperm binding scores, maternal age, previous miscarriage, maternal FSH or AMH concentrations, or paternal sperm concentrations (appendix). There were no differences between groups in the exploratory endpoints of fertilisation and biochemical pregnancy (table 3).

56 serious adverse events were recorded during the study, affecting 31 (2%) of 1386 patients in the PICSI group and 25 (2%) of 1380 patients in the standard ICSI group (appendix). There were two suspected unexpected serious adverse reactions, including one case of hypospadias in the PICSI group and one of achondroplasia in the ICSI group; neither was attributed to treatment.

We did an exploratory analysis to investigate whether trial sampling was representative of national trends and to ascertain the individual contribution of each clinic to the study population. We compared mean term livebirths per embryo transfer, obtained for each site from publicly available HFEA data, with the equivalent values in this study. The mean number of term livebirths per embryo transfer across all sites was 0·26 (SD 0·05) when calculated with HFEA data and 0·23 (0·06) when calculated with trial data (appendix). 26% of couples in the trial were randomised at one site, which had a mean number of livebirths per embryo transfer of 0·23 for both HFEA and trial data. The overall similarity between mean numbers of term livebirths per embryo transfer calculated with HFEA or trial data suggests that neither multiple-site sampling nor relative site contribution affected trial outcomes.

## Discussion

In this multicentre clinical trial, PICSI did not increase the term livebirth rate compared with standard ICSI, and there was no difference between groups in either clinical pregnancy or premature birth. However, we observed a significant reduction in miscarriage with PICSI compared with standard ICSI.

Although our results are largely consistent with those of previous reports,^7^, ^8^, ^9^, ^10^, ^11^ we have provided, for the first time, a robust measure of livebirth following an hyaluronan-based sperm selection procedure. Previous studies^5^, ^7^, ^9^, ^11^, ^13^ of hyaluronan-based sperm selection were inconclusive with respect to livebirth outcome, and with the exception of three studies,^8^, ^9^, ^11^ were not randomised trials or were underpowered. The largest randomised trial^8^ of hyaluronan-based sperm selection before this study did not report livebirth rate. Similar to previous studies,^8^, ^11^ we stratified hyaluronan–sperm binding scores into low-binding and high-binding categories. We did not, however, consider hyaluronan–sperm binding scores in treatment choice or offer PICSI only to those with lower baseline hyaluronan–sperm binding scores. Instead, to make the results of the trial generalisable to all couples undergoing an ICSI procedure, we relied on subgroup analysis of the stratified sample to indicate whether hyaluronan–sperm binding scores affect clinical outcomes of treatment. Whereas we found no association between hyaluronan–sperm binding scores and treatment outcomes, Mokanszki and colleagues^11^ reported a significantly increased livebirth rate in the subgroup with lower (<60%) hyaluronan–sperm binding scores compared with the subgroup with higher (≥60%) scores. However, the sample size of that study was small (n=250), and participants were not randomly assigned to treatment. Given that we saw no effect of hyaluronan–sperm binding scores on outcome, increasing sample size further or restricting the intervention to couples with lower hyaluronan–sperm binding scores to rule it out as a factor in treatment choice seems unnecessary.

Although this study was not powered to investigate miscarriage, we found that the proportion of couples with clinical pregnancy that ended in miscarriage was lower in the PICSI group than in the standard ICSI group, and that the confidence interval for the absolute difference between groups was narrow. This reduction in miscarriage with PICSI has been observed in previous studies.^8^, ^11^ Livebirth is a far more common outcome of the reproductive cycle (natural and assisted) than miscarriage; hence, the confidence interval for the absolute risk difference was wider for livebirth than for miscarriage. This statistical effect helps to explain why the absolute improvement in term livebirth rate, although similar to the absolute reduction in miscarriage, was not significant in this study. The difference of around 2·5% has a greater effect on miscarriage than on term livebirth when presented as a relative difference. Future research should more precisely establish the patient and sample characteristics that underpin the reduction in miscarriage observed in this study, and the mechanism responsible. An independent analysis of trial data to shed light on why PICSI achieved lower miscarriage rates than did standard ICSI in this study will be reported elsewhere.

This study has several strengths. The study had clearly defined outcomes, and all participants and investigators, except for embryologists, were masked to treatment assignment. Moreover, we used locally approved, robust operating standards throughout, which helped minimise any potential logistical and technical limitations resulting from the multicentre design. Despite the approved reduction in power, HABSelect still randomised 2772 couples, which was more than three times the number in the next largest trial of PICSI.^8^ We are therefore confident that the primary outcome was robust. Although embryologists were necessarily not masked to treatment allocation, it is unlikely that they could have biased the assessment of outcomes, which were objectively measured. They could have formed a view about the effectiveness of treatment that might have affected equipoise and influenced recruitment. However, given that allocation concealment was maintained throughout by use of online randomisation after recruitment, it was not possible for embryologists to influence the characteristics of participants allocated to one trial group compared with the other.

Reviews and meta-analyses considering the efficacy of hyaluronan-based sperm selection for ICSI have been equivocal.^5^, ^18^, ^19^ Based on our study design, we can say with confidence that PICSI is not superior to standard ICSI for improving term livebirth rates, despite the reduction in miscarriage. Wider in-service application of PICSI is therefore unjustified at this time.

## Data sharing

Information about HABSelect is already available from the ISRCTN website (ISRCTN99214271), and a summary of the protocol is available. The full, finally approved study protocol, including the statistical analysis plan for the clinical trial and mechanistic analysis of associated trial data, will be made available from the NIHR journals library (https://www.journalslibrary.nihr.ac.uk), alongside their respective reports. The date for full release of these resources, which accompanies publication of the full NIHR HABSelect monograph, is likely to be January, 2019. All reports will be publicly available. A full summary of HABSelect clinical data output, including a data dictionary without any patient identifiable information, will be available in CSV format by request to the chief investigator (DM) as soon as possible after publication of the monograph and subsequent release of the data to the chief investigator.
