# Supplementary material for: Physiological, hyaluronan-selected intracytoplasmic sperm injection for infertility treatment (HABSelect): a parallel, two-group, randomised trial
Source: Lancet. 2019 Feb 2;393(10170):416–22. doi: 10.1016/S0140-6736(18)32989-1 (PMC6409400; doi:10.1016/S0140-6736(18)32989-1)
Supplement: Supplementary appendix [file mmc1.pdf]

THE LANCET

Supplementary appendix

This appendix formed part of the original submission and has been peer reviewed. We post it as supplied by the authors.

Supplement to: Miller D, Pavitt S, Sharma V, et al. Physiological, hyaluronan-selected intracytoplasmic sperm injection for infertility treatment (HABSelect): a parallel, two-group, randomised trial. *Lancet* 2019; **393:** 416–22.

The HABSelect Study Support Team

Chloe	0’Hara
Dima	Abdo
Julia	Andrews
Sara	Barnett
Evelyn	Barrett
Suzanne	Barrett
Stephanie	Bateman
Fiona	Beale
Aysha	Bevan
Debbie	Birch
Sophie	Bird
Jane	Blower
Virginia	Bolton
Cathy	Bowles
Daniel	Breen
Amie	Brunt
Debbie	Bullen
Caroline	Bushby
Yongian	Chen
Sandy	Christiansen
Jane	Clarke
Helen	Clarke
Catherine	Clarkson
Bonnie	Collins
Yaser	Dajani
Merve	Dilgil
Sile	Dunbar
Lucy	Dwyer
Sarah	Fox
Sarah	Fox
Julie	Glanville
Katie	Groves
Geraldine	Hartshorne
Gregory	Horne
Helen	Hunter
Jocelyn	Hunter
Marta	Jansa-Perez
Kirstin	Johnson

The HABSelect Study Support Team

Ben	Lavender
Richard	Lavery
Kayleigh	Lennox
Maneshka	Liyanage
Lilith	Loncke
Rebecca	Lunt
Maureen	Macleod
David	Macmillan
Emma	Maker
Pauline	McBeath
Kevin	McEleney
Caitriona	Meaney
Attia	Mohsen
Therishee	Moodley
Ginny	Mounce
Maria	Nesbitt
Fiona	Newton
Chloe	O'hara
Fiona	Oldman
Adegbile	Oluyemsi
Bijal	Patel
Biserka	Pavlovic
Susan	Pickering
Jenny	Rollason
Alice	Rossie
Jane	Saxton
Allison	Simpson
Michael	Smith
Ivan	Solvas
Arasaratnam	Srikantharajah
Deborah C	Stephenson
Viv	Sutton
Elizabeth	Taylor
Karen	Thompson
Paula	Trinham
Steven	Troup
Karen	Turner
Fiona	Warrander
Kathryn	Warwick

The HABSelect Study Support Team

David	Wells
Kathryn	Whalley
Karolina	Witt
Julie	Wray
Claudette	Wright<br>Yearwood
Charlotte	Martin

Appendix for Physiological, hyaluronan-selected intracytoplasmic sperm injection for infertility treatment (HABSelect): a parallel, two-group, randomised trial

Figure S1· Graphical summary of main clinical outcomes.
Plots of all clinical outcome measures showing Odds Ratios (OR) and 95% confidence intervals (CI), absolute numbers (n), relative proportions (%) and P values.

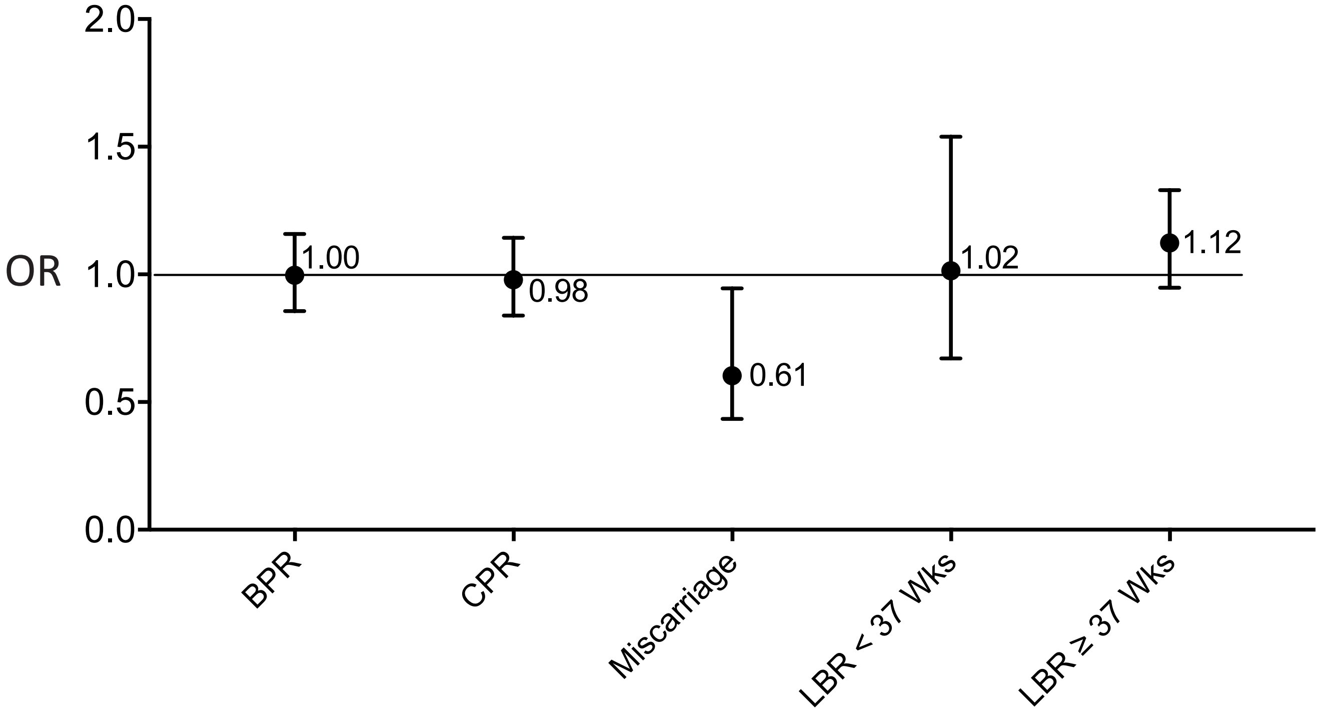

Supplementary Figure 1

Figure S2· Graphical summary of subgroup analysis of trial outcomes·
Plots of Odds Ratios (OR) including 95% confidence Intervals (CI) for A, the primary outcome and B, the miscarriage outcomes are shown.

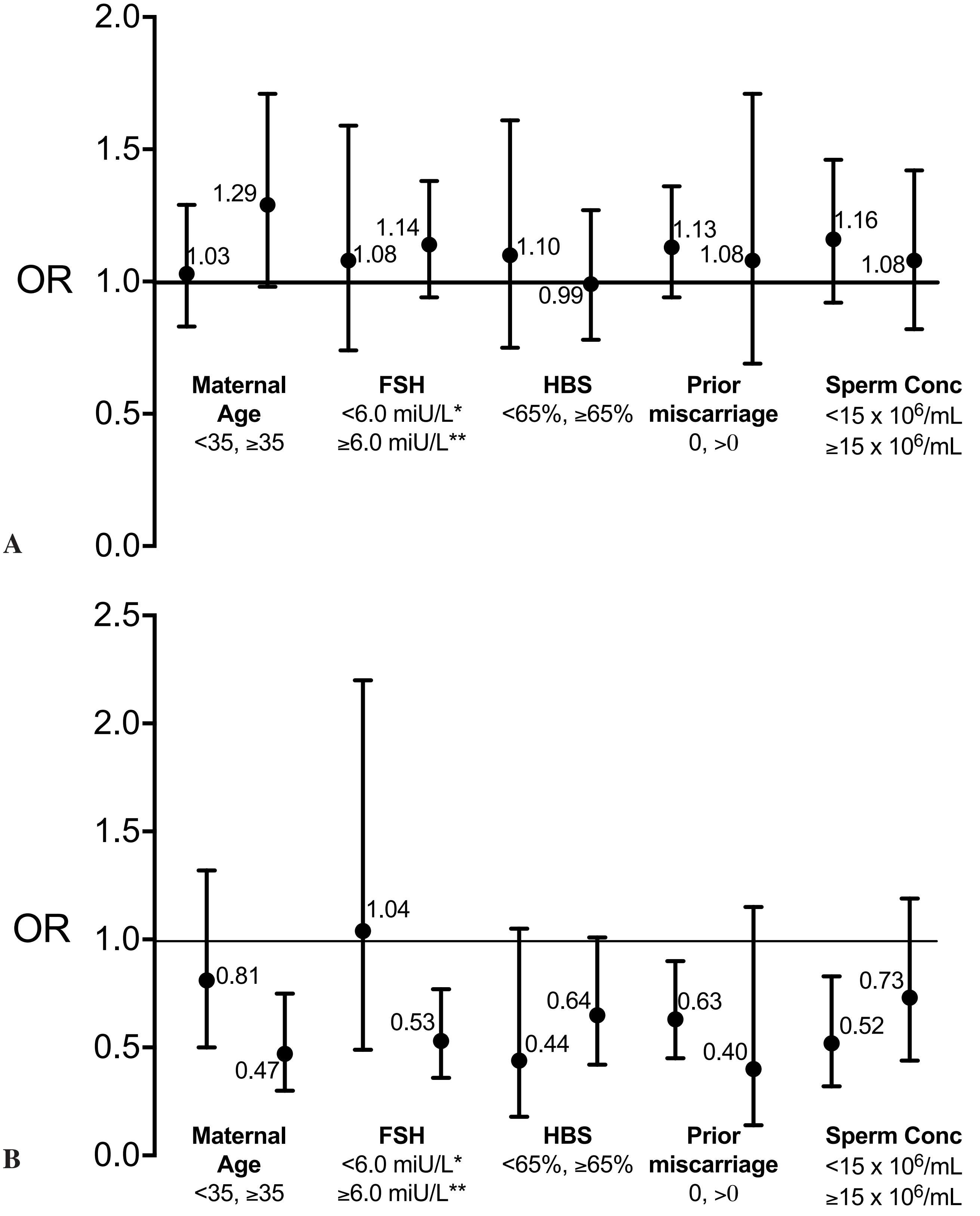

Supplementary Figure 2

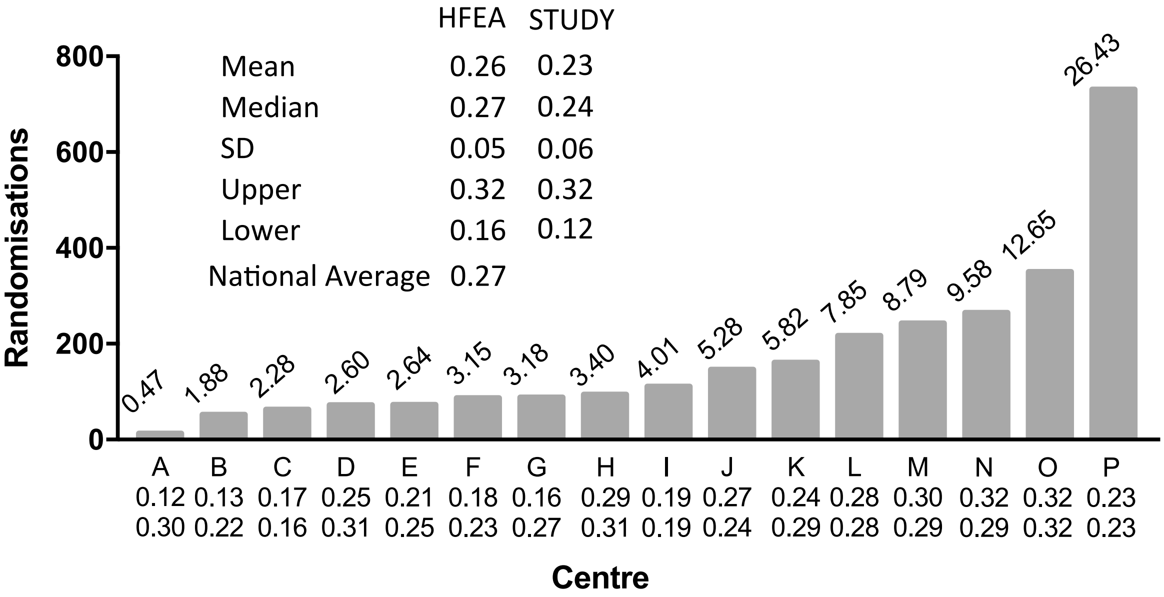

Figure S3. Primary outcome in relation to embryo transfers across participating sites and current HFEA data. The proportions of randomisations (%) contributing to the trial are shown above the bars on a site-by-site basis (A-P). At the base of the bars are the frequencies for term live births per embryo transfer (TLBET) for each site according to trial (upper) and HFEA (lower) data. An accompanying table shows a summary of trial and HFEA TLBET data for all sites. The current (2014-2015) national average frequency for TLBET from all UK centres is 0.27 (https://www.hfea.gov.uk/).

Table S1· Subgroup analyses

A<br>Primary Outcome	Number included in the analysis		Summary		Odds ratio<br>(95% CI)	P-value *
	PICSI<br>N	ICSI<br>N	PICSI<br>N (%)	ICSI<br>N (%)		
**HBA score**						
≤65%	273	254	80 (29·3)	72 (28·3)	1·10 (0·75, 1·61)	0·67
>65%	688	690	178 (25·9)	180 (26·1)	0·99 (0·78, 1·27)	
≤25%	85	74	23 (27·1)	24 (32·4)	0·79 (0·40, 1·58)	0·50
25% > & ≤ 65%	188	180	57 (30·3)	48 (26·7)	1·26 (0·80, 2·01)	
>65%	688	690	178 (25·9)	180 (26·1)	0·99 (0·78, 1·27)	
**Maternal Age**						
< 35	766	755	239 (31·2)	231 (30·6)	1·03 (0·83, 1·29)	0·22
≥ 35	615	616	140 (22·8)	115 (18·7)	1·29 (0·98, 1·71)	
**Previous miscarriage**						
0	1186	1165	327 (27·6)	296 (25·4)	1·13 (0·94, 1·36)	0·86
>0	195	206	52 (26·7)	50 (24·3)	1·08 (0·69, 1·71)	
**FSH level or AMH level where FSH not tested**						
< 6·0 miU/L (< 17·0 pmol/L for AMH)	291	272	78 (26·8)	68 (25·0)	1·08 (0·74, 1·59)	0·82
≥ 6·0 miU/L (≥ 17·0 pmol/L for AMH)	1090	1099	301 (27·6)	278 (25·3)	1·14 (0·94, 1·38)	
**Sperm concentration**						
<15x10^6/ml	777	763	225 (29·0)	196 (25·7)	1·16 (0·92, 1·46)	0·71
≥ 15x10^6/ml	553	566	141 (25·5)	140 (24·7)	1·08 (0·82, 1·42)	

B<br>Miscarriage Outcome	Number included in the analysis		Summary			
	**PICSI**<br>**N**	**ICSI**<br>**N**	**PICSI**<br>**N (%)**	**ICSI**<br>**N (%)**	**Odds ratio**<br>**(95% CI)**	**P-value***
**HBA score**						
≤65%	273	254	8 (2·9)	16 (6·3)	0·44 (0·18, 1·05)	0·43
>65%	688	690	35 (5·1)	52 (7·5)	0·65 (0·42, 1·01)	
≤25%	85	74	1 (1·2)	2 (2·7)	0·42 (0·04, 4·71)	0·75
25% > & ≤ 65%	188	180	7 (3·7)	14 (7·8)	0·45 (0·18, 1·15)	
>65%	688	690	35 (5·1)	52 (7·5)	0·65 (0·42, 1·01)	
**Maternal Age**						
< 35	766	755	31 (4·0)	38 (5·0)	0·81 (0·50, 1·32)	0·11
≥ 35	615	616	29 (4·7)	58 (9·4)	0·47 (0·30, 0·75)	
**Previous miscarriage**						
0	1186	1165	55 (4·6)	83 (7·1)	0·63 (0·45, 0·90)	0·42
>0	195	206	5 (2·6)	13 (6·3)	0·40 (0·14, 1·15)	
**FSH level or AMH level where FSH not tested**						
< 6·0 miU/L (< 17·0 pmol/L for AMH)	291	272	15 (5·2)	14 (5·1)	1·04 (0·49, 2·20)	0·12
≥ 6·0 miU/L (≥ 17·0 pmol/L for AMH)	1090	1099	45 (4·1)	82 (7·5)	0·53 (0·36, 0·77)	
**Sperm concentration**						
<15x10^6/ml	777	763	28 (3·6)	53 (6·9)	0·52 (0·32, 0·83)	0·33
≥ 15x10^6/ml	553	566	29 (5·2)	39 (6·9)	0·73 (0·44, 1·19)	

Table S1· Subgroup analyses of primary and miscarriage outcomes.

Subgroup analysis for primary **(A)** and miscarriage (**B**) outcomes are shown· *P-values are for the interaction term between the subgroup variable and the treatment variable (for two levels, the p-value is from a likelihood ratio test including or not including the interaction term).

Table S2· Categories of serious adverse events (SAE)

Treatment	N	*Congenital Abnormality	Neonatal Death	Maternal death	Misc Other
PICSI	31	10	2	0	19
ICSI	25	9	0	1	15
Total	56	19	2	1	34

Table S2 Categorisation of Serious Adverse Events (SAE) in HABSelect.

*Two SAEs were congenital abnormalities recorded as Serious Unexpected Serious Adverse Reactions (SUSARs); a case of achondroplasia and a case of hypospadias at two different centres. Arguably, one and probably both of these events were not SUSAR according to accepted UK definitions. Septicaemia arising from ruptured membranes and subsequent infection caused maternal death in one case.

HABSelect

Statistical Analysis Plan

Version: 2.0
Date: 17/08/2017

Person(s) contributing to the analysis plan	
Name(s) and position(s)	Gordon Forbes, Lee Beresford – Trial Statisticians<br>Dr Richard Hooper – Senior Trial Statistician<br>Dr David Miller – Chief Investigator<br>Dr Steve Roberts – Independent statistician
Authorisation	
Position	**Chief or principal investigator**
Name	Dr David Miller
Signature	
Date	21/08/17
Position	**Senior trial statistician**
Name	Dr Richard Hooper
Signature	
Date	21 Aug 2017
Position	**Independent statistician***
Name	Dr Steve Roberts
Tick once reviewed	✓
Date	17/08/2017

*This will be the Trial Steering Committee (TSC) Statistician

Changes to SAP from version 1.0

- Appendix 1 amended to provide more details on how the trial outcomes and other derived variables will be calculated. Detail was added on when an outcome can be classed as negative and when it will be considered missing.

Abstract

Background: *Intra-Cytoplasmic Sperm Injection (ICSI) is a procedure in Assisted Reproduction Technologies, where, instead of the egg being the final arbiter for selection, the 'right' single sperm is selected for each egg by the embryologist. Clinically relevant studies suggest that using a hyaluronic acid binding based selection procedure (using hyaluronic acid coated plates) increases live birth rates as well as having a number of other positive effects. The HABSelect trial aims to evaluate the effectiveness of this sperm selection procedure, versus sperm selected on a conventional visual basis prior to ICSI., on increasing live birth rate. Other secondary measures will also be evaluated.*

Methods/Design: *The trial is a parallel group, two arm, multicentre, blinded, randomised controlled efficacy clinical trial with mechanistic evaluation. A total of 3266 participants will be randomised. The primary outcome is live birth rate beyond 37 weeks gestation. Secondary outcome measures are also presented and the proposed statistical analysis for all outcome measures are outlined in detail in this paper.*

Conclusion: *The HABSelect trial investigates the effect of using a hyaluronic acid binding based sperm selection approach in Intra-Cytoplasmic Sperm Injection procedures. Clinical outcomes from the HABSelect trial will be analysed according to this pre-specified statistical analysis plan.*

Trial Registration: *HABSelect is registered in ISRCTN under ISRCTN99214271*

Keywords: *HABSelect Trial; Intra-Cytoplasmic Sperm Injection; Live Birth Rate; Polyvinylpyrrolidone; Hyaluronic Acid; Hyaluronan; Sperm Selection; Assisted Reproduction Technologies; Clinical Pregnancy; Miscarriage; Male Fertility; Randomised Controlled Trial; Statistical Analysis Plan*

Introduction

In 2008, almost 40,000 couples in the UK alone were treated with assisted reproduction technologies (ART), compromising of 50,687 in vitro fertilisation (IVF) cycles. This number is set to rise in the coming years (1). Currently live birth rates for ART are at an average of 24% per treatment cycle, although live birth rates per couple are higher at 32%, because couples normally receive an average of approximately 1.3 treatment cycles. While it is estimated that more than two thirds of naturally conceived pregnancies end in failure, the limit for improvements in live birth rates following ART may not have been reached.

For all ART procedures including intra-cytoplasmic sperm injection (ICSI), the embryologist seeks to use the best sperm available. Selection is aided by sperm ‘washing’ techniques using density gradient configuration (DGC) that can enrich for sperm with high motility and good morphology (WHO Manual, 2010) (2). In contrast with standard IVF, where the egg is the final arbiter for selection, ICSI is dependent on the relatively subjective judgment of the andrologist or embryologist to choose the ‘right’ single sperm for each egg.

Various studies have shown clear inverse relationships between DNA damaged sperm in the ejaculate and clinical pregnancy or live birth rates in standard IVF, but this relationship is less obvious with ICSI cycles (3). We recently reported reductions in levels of sperm DNA fragmentation following density gradient washing of semen and while the values from washed semen were reduced, they were still over twice as high in the non-pregnant (approximately 50%) versus pregnant (approximately 23%) cohorts. These and other data suggest that sperm with poor DNA quality persist in washed sperm preparations from fertile and infertile men (4-13) and unlike IVF, where there is a natural selection by the egg, ICSI could be particularly vulnerable to a poor choice of sperm. By eliminating abnormal sperm from the sample preparation for ICSI, success rates should rise accordingly.

It has been shown that immature sperm with higher rates of DNA damage have a dysfunctional ability to bind to hyaluronic acid (14, 15), which is the major glycosaminoglycan secretion of the cervix. In many clinics, polyvinylpyrrolidone (PVP) is normally used to slow sperm down sufficiently for capture in ICSI procedures (standard-ICSI). However, clinically relevant studies (16, 17) suggest that using hyaluronic acid-selected (using hyaluronic acid coated plates) Physiological Intra-Cytoplasmic Sperm Injection (PICSI) instead of standard-ICSI increases live birth rate and the numbers of grade one embryos.. There is also strong evidence that PICSI reduces early pregnancy failure (17). The HABSelect (Hyaluronic Acid Binding Sperm Selection) trial aims to confirm this by comparing the use of a HA (hyaluronic acid) selection step prior to ICSI (PICSI) with standard-ICSI for treatment of male fertility for the treatment of male infertility in a rigorous randomised controlled efficacy trial. A successful conclusion of the study will help

provide a more consistent and efficient procedure for ICSI sperm selection which complies with and extends on the National Institute of Clinical Excellence's (NICE) recently called review on fertility guidance (18).

This paper describes the statistical analysis plan (SAP) for the clinical outcomes of the HABSelect trial. A mechanistic evaluation of the action of hyaluronic sperm selection will also be undertaken and included within the final report. However, planning for this aspect of the trial will be documented separately and only analysis of clinical outcomes are specified within this document. In accordance with good clinical practice, this SAP was drafted without any knowledge of the outcomes by the investigators and this blinding will not be broken before the analysis plan is finalised and signed off.

Trial Overview and Design

Overview: The HABSelect trial is a parallel group two arm multi-centre blinded randomised controlled efficacy clinical trial, with mechanistic evaluation, comparing the use a HA (hyaluronic acid) selection step prior to physiological ICSI (PICSI) with standard-ICSI for treatment of male infertility, with the objective of increasing live birth outcomes and reducing miscarriage rates.

Study Population: The study population for randomisation represents couples undergoing ICSI procedure, with the ability to provide informed consent. The following inclusion criteria are also imposed:

Women:

- BMI: 19.0 – 35.0 kg/m^2
- FSH level: 3.0 – 20.0 miU/ml and / or AMH ≥ 1.5 pmol/L
- Age: 18-43

Men:

- Age: 18 – 55
- Able to produce freshly ejaculated sperm for the treatment cycle

The exclusion criteria for the trial are as follows:

- Couples who have not consented prior to ICSI will be ineligible
- Couples using non-ejaculated sperm
- Couples using donor gametes
- Men with vasectomy reversal; cancer treatment involving any chemotherapy and / or radiotherapy in the past two years
- Previous participation in the HABSelect trial
- Split IVF / ICSI procedures

- If both FSH and AMH are tested and either of them falls outside the accepted range

There are 15 participating centres. Recruitment rates will be monitored and optional additional centres may be added as required. Centres will be IVF licensed hospitals and must be able to provide appointments in a dedicated clinic in which to see participants.

Consent: Written informed consent will be obtained by the principal investigator, or by another suitably qualified member of the trial team. This will compromise of a written consent form, and will be obtained for each couple before enrolment in the trial. Patients have the right to refuse consent and / or withdraw from the study at any time without giving reasons and without prejudicing any further treatment.

Randomisation Procedures: Couples are randomised in a ratio of 1:1 to either intervention (PICSI) or non-intervention (standard-ICSI) arms. Randomisation is stratified by four criteria

- Maternal age (<35, ≥35)
- Paternal age (<35, ≥35)
- Number of previous miscarriages (0,1-2, >2)
- Hormonal indicator of ovarian reserve: FSH (<6.0, ≥6.0 miU/ml) or AMH (<17.0, ≥17.0 pmol/L) when FSH is not available.

Minimisation factors are balanced separately within each centre.

Treatment Procedures: In the non-interventional arm (standard-ICSI) density gradient washed and prepared motile sperm and visually selected for ICSI with the aid of an inverted microscope. In the interventional arm (PICSI) exactly the same procedure is carried out except that the washed and prepared motile sperm are allowed to interact with and become attached to a specifically prepared HA-coated surface beforehand. The HA-selection process is henceforward referred to as PICSI.

Treatment Blinding: Participants, clinical care providers in IVF licensed units, maternity & neonatal wards and research nurses responsible for participants follow up will be blinded to treatment allocation. The only unblinded group at study sites is going to be the embryologist who performs the PICSI / standard-ICSI procedure, hyaluronic acid binding scoring (HBS) and randomisation. Those within the PCTU who will remain unblinded will be the study data manager and independent statistician, who will prepare reports for the Data Monitoring and Ethics Committee (DMEC).

An anticipated CONSORT flow chart for the trial is displayed in Figure 1.

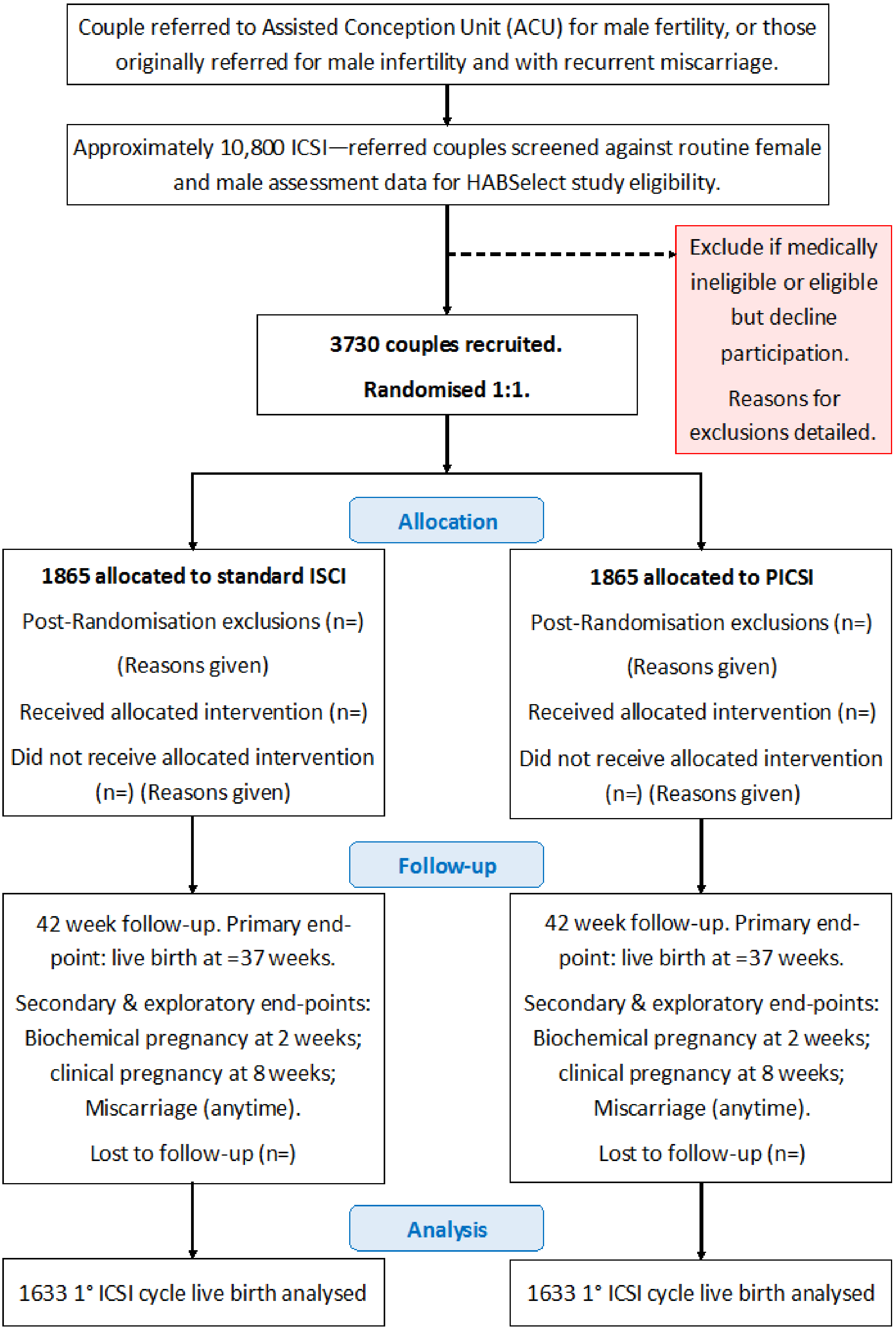
Couple referred to Assisted Conception Unit (ACU) for male fertility, or those originally referred for male infertility and with recurrent miscarriage.
Approximately 10,800 ICSI—referred couples screened against routine female and male assessment data for HABSelect study eligibility.
Exclude if medically ineligible or eligible but decline participation.
Reasons for exclusions detailed.
3730 couples recruited.
Randomised 1:1.
Allocation
1865 allocated to standard ISCI
Post-Randomisation exclusions (n=)
(Reasons given)
Received allocated intervention (n=)
Did not receive allocated intervention (n=) (Reasons given)
1865 allocated to PICSI
Post-Randomisation exclusions (n=)
(Reasons given)
Received allocated intervention (n=)
Did not receive allocated intervention (n=) (Reasons given)
Follow-up
42 week follow-up. Primary end-point: live birth at =37 weeks.
Secondary & exploratory end-points: Biochemical pregnancy at 2 weeks; clinical pregnancy at 8 weeks; Miscarriage (anytime).
Lost to follow-up (n=)
42 week follow-up. Primary end-point: live birth at =37 weeks.
Secondary & exploratory end-points: Biochemical pregnancy at 2 weeks; clinical pregnancy at 8 weeks; Miscarriage (anytime).
Lost to follow-up (n=)
Analysis
1633 1° ICSI cycle live birth analysed
1633 1° ICSI cycle live birth analysed

Figure 1: Anticipated CONSORT flow-chart for HABSelect.

Objectives / Outcome Measures

The main aims of HABSelect are to

1. Show that a hyaluronic acid binding step in an assisted reproduction setting can significantly improve live birth rate
2. Assess how the chromatin status of HA-selected versus conventionally recovered sperm corresponds with HBS, clinical pregnancy, live birth rate and pregnancy loss.

Primary Objective: To determine the efficacy of PICSI versus standard-ICSI in a rigorous randomised controlled clinical trial of participants where the primary outcome measure will be live birth rate ≥ 37 weeks gestation after first fresh embryo transfer.

Secondary Objectives: To determine the impact of PISCI versus standard ICSI on:

- Increasing clinical pregnancy rate based on detection of fetal heartbeat or presence of fetal sac at 6-9 weeks gestation
- Reducing miscarriage rate defined as pregnancy loss after confirmation of clinical pregnancy
- Increasing live birth rate at <37 weeks gestation

Primary outcome measure: Live birth at ≥ 37 weeks gestation following the first fresh PICSI/ICSI treatment.

Secondary outcome measures:

- Clinical pregnancy rate based on detection of a fetal heartbeat or the presence of fetal sac at 6-9 weeks gestation
- Miscarriage, defined as pregnancy loss after confirmation of clinical pregnancy
- Live birth <37 weeks gestation

Sample Size

From our study feasibility audit data, we estimate that around 4663 men per annum will be eligible for an ICSI procedure across all 10 participating centres. Given our broad inclusion criteria, we conservatively expect that 40% of the couples to be eligible and willing to consent to the study i.e. 3730 over 21 months. Trial recruitment

is based on pro rata targets at each of the participating sites of the Human Fertilisation and Embryology Authority (HFEA) data and the need to recruit at least 3266 couples into the trial to detect a 5% improvement in clinical efficacy with a power of 90%.

For PISCI, average improvements in live birth rate per treatment cycle are likely to be 7.5% based on maternal age and paternal semen profile (16, 17, 19). Older women (≥37) are of particular interest to us because their eggs may have a decreased capacity for repairing sperm DNA damage in their older partners (20). Lower and higher improvement scores among younger and older women are likely, respectively. Assuming 5% for the former and 15% for the latter, live birth rate will rise from 32.7% to 37.7% (3826 treatment cycles) and 19.3% to 34.3% (358 treatment cycles) in women <35 and women >37, respectively. Because they lie between the more fertile younger and less fertile older age groups, improvements for women aged between 35 and 37 are likely to reflect that of women of all ages, at 7.5%. We assume that miscarriage rate will be inversely correlated with live birth rate and therefore it is unnecessary to repower for it. Clearly, we shall have sufficient recruitment into the study to test outcomes in relation to HBS predictions and parental age. However, lower improvement rates (among younger couples in particular) will incur lower accuracies unless power is relaxed to 80%. Improvement among older women is certainly testable, as those >37 now account for almost 30% of ART procedures, providing 1007 women for the study.

A 10% loss to follow-up (the envisaged worst-case scenario) will still ensure outcomes for 3357 primary treatment cycles, which is sufficient to power the study at even 5% improvement per couples undergoing a fresh ICSI treatment cycle. It is anticipated, however, that compliance with follow-up will be high, given the lateness of randomisation and the routine nature of collecting pregnancy outcome data in this population (refer to anticipated CONSORT flow-diagram – Figure 1).

General Statistical Considerations

All analyses will be intention-to-treat (ITT): all couples randomised, with the exception of those enrolled in error or for who consent was not obtained, with a recorded outcome will be included in the analysis, and analysed according to the arm to which they were randomised (21). Every attempt will be made to gather data on all women randomised, irrespective of compliance with the treatment protocol.

Post Randomisation Exclusions: Certain exclusions will be made for the analysis, post-randomisation. These will be all couples who were enrolled in error or because consent was not obtained. Women whose BMI calculated baseline height and weight to be greater than or equal to 18 or less than or equal to 36 will be included in the

trial to allow for rounding errors in BMI. Women with BMI below 18 or above 36 will be excluded from the trial as they do not meet the eligibility criteria and will be considered to be enrolled in error. Women who are found not to meet any other eligibility criteria will be excluded from the analysis. Women who withdraw their consent will still be analysed unless it was specified by them that their data should not be used, in which case the data will be excluded from the trial analysis.

For the primary analysis, all secondary analysis and sensitivity analysis we will report an odds ratio for the effect of the intervention with a 95% confidence interval and two sided P-value.

All investigators will remain blinded prior to the final analysis so as not to bias the analysis and interpretation of results. An independent statistician employed by the PCTU provided the DMEC with unblinded summaries and reports using computer code provided by the study statistician.

Stata version 12 or higher will be used to code and produce statistical analysis, but other software such as R may be used if appropriate.

Missing data: Every attempt will be made to collect full follow up data on all couples and it is anticipated that missing data will be minimal. The ‘missingness’ in outcome and baseline data will be summarised, with breakdowns of ‘missingness’ by trial arm, for example. Where baseline covariates are missing, mean imputation will be used for continuous covariates and a missing indicator will be used for categorical variables. Note that epidemiological arguments against the use of a missing indicator do not apply in randomised trials (22).

If any outcome data are missing we will analyse only those with outcome data, adjusting for baseline covariates. This approach is unbiased if the outcome is ‘Missing At Random’ (MAR) i.e. ‘missingness’ for the outcome is related to the observed covariates. If ‘missingness’ in the primary outcome is >5% then a sensitivity analysis will be conducted to explore the MAR assumption. In this case, a pattern-mixture model estimated by a mean score approach will be adopted (23). We will model the primary outcome using logistic regression, adjusting for maternal age, paternal age, number of previous miscarriages, and hormonal indicator of ovarian reserve in the same way as the primary analysis. Centre will be accounted for using clustered sandwich variance estimator. We will vary the informative ‘missingness’ odds ratio between 1/3 and 3, i.e. the probability of a missing outcome being a live birth ≥ 37 weeks is between 1/3 and 3 times as likely as an observed outcome.

Statistical Analysis

Evaluation of Demographics and Baseline Covariates: Numbers of couples who are eligible, recruited and followed up will be recorded in a CONSORT flow-chart. Baseline characteristics of couples in each arm will be summarised with counts (percentages) for categorical variables and median with interquartile range (IQR) for continuous variables. Mean and standard deviation may also be used to summarise continuous variables where appropriate.

Primary Analysis: The primary outcome measure is the proportion of women randomised who experience a live birth ≥ 37 weeks. This proportion has as its denominator the number of women who are randomised to either intervention (PICSI) or non-intervention (standard-ICSI) with data recorded and as its numerator the number of women who conceive and proceed to have a live birth ≥ 37 weeks as a result of their first fresh ICSI cycle. Please see Appendix 1 for a description of the variables to be used in the derivation of the primary outcome.

Differences in the proportion between treatment arms will be assessed using mixed effects logistic regression model. The analysis will adjust for the minimisation variables: maternal age, paternal age, number of previous miscarriages, and hormonal indicator of ovarian reserve. Centre will be included as a random intercept. Maternal age and paternal age will be adjusted for using restricted cubic splines with three knots (knot locations based on Harrell's recommendations) (24, 25). Number of previous miscarriages, and hormonal indicator of ovarian reserve will be adjusted for as categorical variables. Number of previous miscarriages will have three categories 0,1-2, >2. Hormonal indicator of ovarian will have two categories FSH <6.0, ≥6.0 miU/ml or AMH <17.0, ≥17.0 pmol/L when FSH is not available.

Sensitivity Analyses: If there is evidence that the secondary outcome clinical pregnancy rate differs between the treatment arms, then as a sensitivity analysis, the primary outcome will be reanalysed taking only women who experience a clinical pregnancy as the denominator. This analysis will be carried out using the same mixed effects logistic regression described for the primary analysis with an analysis population of all couples included in the primary analysis who experienced a clinical pregnancy.

As an additional sensitivity analysis, the primary outcome will be reanalysed using a mixed effect logistic regression, including centre as a random intercept, adjusting for the minimisation variables as described in the primary analysis model and adjusting for additional factors believed to be potentially prognostic or associated with the outcome. Additional factors to be adjusted for in this analysis are:

- Female partner BMI (adjusted for using restricted cubic splines with three knots (knot locations based on Harrell's recommendations) (24, 25))

- Female partner ethnicity (adjusted for using four categories: White / Asian or Asian British / Black or Black British / Other)
- History of previous pregnancy (adjusted for using two categories: yes/no)
- Female partner smoking status (adjusted for using two categories: current smoker/not current smoker)
- Stimulation treatment (adjusted for using three categories: long agonist / short agonist / antagonist)

In all cases, results of the primary analysis will be given more weight than those of any sensitivity analyses.

Subgroup Analysis: The following subgroup analyses will be performed for the primary outcome:

- Analysis of treatment effect by HBS (high (>65%) versus low (≤65%))
- Analysis of treatment effect by maternal age (<35 years verses ≥35)
- Analysis of treatment effect by number of previous miscarriages (0 versus >0)
- Analysis of treatment effect by Follicle stimulating hormone (FSH) hormone level (<6.0miU/ml versus ≥ 6.0miU/ml) or Anti-Mullerian Hormone (AMH) hormone level (<17pmol/L versus ≥ pmol/L) where FSH testing is not available
- Analysis of treatment effect by sperm concentration (<15mml versus ≥ 15mml)

We may also analyse treatment effect by a very low HBS sub-group, depending on numbers available (≤25%) versus a low HBS sub-group (>25%, ≤65%)

The subgroup analysis will be carried out using the same model as the primary analysis including a subgroup by treatment interaction term. Subgroup specific estimates (for planned and exploratory analyses) will be reported with 95% confidence intervals and displayed graphically. All subgroup analyses will be hypothesis generating and findings will be treated with caution. Hence there will be no corrections made for the issue of multiplicity.

Secondary Analysis: The secondary outcomes: clinical pregnancy rate, miscarriage, and live birth <37 weeks gestation will be analysed using mixed effects logistic regression models. Each secondary outcome will have as its denominator the number of women who are randomised to either intervention (PICSI) or non-intervention (standard-ICSI) with data recorded for the outcome. All secondary

analyses will include centre as a random intercept and adjust for maternal age, paternal age, number of previous miscarriages, and hormonal indicator of ovarian reserve. Maternal age and paternal age will be adjusted for using restricted cubic splines with three knots (knot locations based on Harrell's recommendations) (24, 25). Number of previous miscarriages, and hormonal indicator of ovarian reserve will be adjusted for as categorical variables. Number of previous miscarriages will have three categories 0,1-2, >2. Hormonal indicator of ovarian will have two categories FSH <6.0, ≥6.0 miU/ml or AMH <17.0, ≥17.0 pmol/L when FSH is not available.

Other Data summaries

Serious adverse events (SAEs)

The number of SAEs will be summarised for each treatment group.

Other follow up data and intermediate outcomes

Follow up data collected which is not for primary or secondary outcomes will be summarised by treatment group by the mean and standard deviation or median and interquartile range for continuous variables, and the number and percent for categorical variables. Differences between groups will not be presented and no statistical tests will be performed on this data. This will include summaries of:

- Oocytes collected (per couple)
- Fertilisation rate (number of two pronuclei stage eggs per injected egg)
- Number of embryos created
- Number of single and double embryo transfers
- Biochemical pregnancy rate (bHGC test)
- Multiple pregnancy rate
- Multiple birth rate

Mechanistic Evaluation

As indicated in the protocol, a mechanistic evaluation will also be undertaken. Planning for these analyses will be documented separately.

Conclusion

With this SAP we present the analyses that will be published in the primary publication for the clinical aspect of the trial. By agreeing this SAP prior to unblinding of any investigators, we avoid any bias that may arise from knowledge of outcome and data-driven results.

The aim of the HABSelect study is to compare the use of PICSI to standard-ISCI procedures for treatment of male fertility. With the publication of this paper pre-specifying the analyses to be used, we hope that the results from the HABSelect trial will be as transparent as possible.

Abbreviations

IVF: In Vitro Fertilisation; ART: Assisted Reproduction Technologies; ICSI: Intra-Cytoplasmic Sperm Injection; DGC: Density Gradient Configuration; PVP: Polyvinylpyrrolidone; PISCI: PISCI (hyaluronic acid coated plated) Selected Intra-Cytoplasmic Sperm Injection; HABSelect: Hyaluronic Acid Binding Sperm Selection; NICE: National Institute of Clinical Excellence; SAP: Statistical Analysis Plan; HBS: Hyaluronic Acid Binding Score; DMEC: Data Monitoring and Ethics Committee; HFEA: Human Fertilisation and Embryology Authority; ITT: Intention To Treat; MAR: Missing At Random; SD: Standard Deviation; IQR: Inter-quartile Range; FSH: Follicle Stimulating Hormone; AMH: Anti-Mullerian Hormone

References

1. European Society of Human Reproduction and Embryology. Art Fact Sheet [Available from: http://www.eshre.eu/sitecore/content/Home/Guidelines%20and%20Legal/ART%20fact%20sheet.
2. WHO ed. W.H. Organization. 2010.
3. Zini A, Bielecki R, Phang D, Zenzes MT. Correlations between two markers of sperm DNA integrity, DNA denaturation and DNA fragmentation, in fertile and infertile men. Fertility and Sterility. 2001;75(4):674-7.
4. Simon L, Lutton D, McManus J, Lewis SEM. Sperm DNA damage measured by the alkaline Comet assay as an independent predictor of male infertility and in vitro fertilization success. Fertility and Sterility. 2011;95(2):652-7.
5. Sousa APM, Tavares RS, de la Calle JFV, Figueiredo H, Almeida V, Almeida-Santos T, et al. Dual use of Diff-Quik-like stains for the simultaneous evaluation of human sperm morphology and chromatin status. Hum Reprod. 2009;24(1):28-36.
6. Micinski P, Pawlicki K, Wielgus E, Bochenek M, Tworkowska I. The sperm chromatin structure assay (SCSA) as prognostic factor in IVF/ICSI program. Reprod Biol. 2009;9(1):65-70.
7. Gu LJ, Chen ZW, Chen ZJ, Xu JF, Li M. Sperm chromatin anomalies have an adverse effect on the outcome of conventional in vitro fertilization: a study with strictly controlled external factors. Fertility and Sterility. 2009;92(4):1344-6.
8. Zini A, Boman JM, Belzile E, Ciampi A. Sperm DNA damage is associated with an increased risk of pregnancy loss after IVF and ICSI: systematic review and meta-analysis. Hum Reprod. 2008;23(12):2663-8.
9. Lewis SEM, Agbaje I, Alvarez J. Sperm DNA tests as useful adjuncts to semen analysis. Syst Biol Reprod Mec. 2008;54(3):111-25.

10. Bhattacharya SM. Association of various sperm parameters with unexplained repeated early pregnancy loss - which is most important? Int Urol Nephrol. 2008;40(2):391-5.
11. Bakos HW, Thompson JG, Feil D, Lane M. Sperm DNA damage is associated with assisted reproductive technology pregnancy. Int J Androl. 2008;31(5):518-26.
12. Zini A, Libman J. Sperm DNA damage: clinical significance in the era of assisted reproduction. Can Med Assoc J. 2006;175(5):495-500.
13. Lewis SEM, Aitken RJ. DNA damage to spermatozoa has impacts on fertilization and pregnancy. Cell Tissue Res. 2005;322(1):33-41.
14. Huszar G, Patrizio P, Vigue L, Willets M, Wilker C, Adhoot D, et al. Cytoplasmic extrusion and the switch from creatine kinase B to M isoform are completed by the commencement of epididymal transport in human and stallion spermatozoa. J Androl. 1998;19(1):11-20.
15. Moretti E, Gergely A, Zeyneloglu HB, Ward P, Ward D, Baccetti B, et al. Relationship among head size, morphology and chromosome structure in human spermatozoa. Fertility and Sterility. 1997:P138-P.
16. Parmegiani L, Cognigni GE, Ciampaglia W, Pocognoli P, Marchi F, Filicori M. Efficiency of hyaluronic acid (HA) sperm selection. J Assist Reprod Gen. 2010;27(1):13-6.
17. Worrilow KC, Eid S, Woodhouse D, Perloe M, Smith S, Witmyer J, et al. Use of hyaluronan in the selection of sperm for intracytoplasmic sperm injection (ICSI): significant improvement in clinical outcomesumulticenter, double-blinded and randomized controlled trial. Hum Reprod. 2013;28(2):306-14.
18. National Institute of Clinical Excellence. Review of Fertlity Guidance.
19. Prinosilova P, Kruger T, Sati L, Ozkavukcu S, Vigue L, Kovanci E, et al. Selectivity of hyaluronic acid binding for spermatozoa with normal Tygerberg strict morphology. Reprod Biomed Online. 2009;18(2):177-83.
20. Meseguer M, Santiso R, Garrido N, Garcia-Herrero S, Remohi J, Fernandez JL. Effect of sperm DNA fragmentation on pregnancy outcome depends on oocyte quality. Fertil Steril. 2011;95(1):124-8.
21. White IR, Horton NJ, Carpenter J, Pocock SJ. Strategy for intention to treat analysis in randomised trials with missing outcome data. BMJ. 2011;342:d40.
22. White IR, Thompson SG. Adjusting for partially missing baseline measurements in randomized trials. Stat Med. 2005;24(7):993-1007.
23. White IR. Sensitivity analysis for randomised trials with missing outcome data: UK Stata Users' Group; 2011 [Available from: http://www.stata.com/meeting/uk11/abstracts/UK11_White.pdf.
24. Kahan BC, Rushton H, Morris TP, Daniel RM. A comparison of methods to adjust for continuous covariates in the analysis of randomised trials. BMC Med Res Methodol. 2016;16:42.
25. Harrell FE. Regression Modeling Strategies: With Applications to Linear Models, Logistic Regression, and Survival Analysis. New York: Springer; 2001.

Appendix 1: Derived outcomes

Baseline and pre-intervention data

Maternal and paternal age

- Maternal and paternal age will be calculated as the difference between the date of birth provided in the baseline CRF and the date of randomisation.

Maternal and paternal body mass index (BMI)

- BMI will be calculated as weight(kg)/height(m)^2.

Anti-Mullerian hormone (AMH)

- AMH is collected with two sets of units: pmol/l and ng/ml. We will convert between the two using the conversion factor 1 pmol/ = 0.14 ng/ml.

Hyaluronan binding score (HBS)

- Where two measurements are taken the Hyaluronan binding score will be the mean of the two measurements.
- Where only one measurement was taken this will be the Hyaluronan binding score.
- Hyaluronan binding score score will be missing if no measurements were taken.

A note on study completion

For all couples in the HABSelect study a data was collected on whether the couple "completed the study". If a couple is recorded as completing the study this indicates that follow up for pregnancy outcome data was completed.

The HABselect CRFs were structured in such a way that if a biochemical pregnancy test or clinical pregnancy test was not carried out then no data would be entered for that test or for any fetus outcomes. Similarly if no clinical pregnancy occurred no data would be entered for fetus outcomes.

When data is not entered for a biochemical pregnancy test or clinical pregnancy test and the couple are recorded as completing the study we know that a pregnancy did not occur.

Where couples are recorded as not completing the study additional data is collected on the reason for pregnancy data not being collected. Where the reason given for not completing the study does not exclude the possibility of pregnancy, for example couple switched to IVF or couple lost to follow up, then outcomes will be considered missing where no data is recorded to indicate otherwise. In these cases further steps will be taken to complete study follow up to minimise the amount of missing data.

Where the reason given excludes the possibility of pregnancy, for example "all embryos frozen due to risk of ovarian hyper stimulation syndrome" then pregnancy outcomes will be considered negative.

The list of reasons for not completing the study will be reviewed by the chief investigator prior to unblinding and classified as either excluding the possibility of pregnancy or not excluding the possibility of pregnancy.

Intermediate outcomes

Biochemical pregnancy

If a biochemical pregnancy test was carried out biochemical pregnancy will be positive if any of the following occur:

- First biochemical pregnancy test is positive.
- First biochemical pregnancy test is inconclusive and second test biochemical pregnancy is positive.

If a biochemical pregnancy test was carried out biochemical pregnancy will be negative if any of the following occur:

- The first biochemical pregnancy test is negative.
- The first biochemical pregnancy test is inconclusive and second biochemical pregnancy test is recorded as negative.
- The first biochemical pregnancy test is inconclusive, no results are recorded from a second biochemical pregnancy test and participant is recorded as "completing the study" (see note above).

If a biochemical pregnancy test was carried out biochemical pregnancy will be missing if **all** of the following occur:

- The first biochemical pregnancy test is inconclusive.
- The second test not completed.
- Participant is recorded as "not completing the study". Reason given for not completing the study does not indicate no biochemical pregnancy. Reasons for not completing the study will be reviewed by the chief investigator prior to unblinding of the treatment allocations.

If no results from biochemical pregnancy tests are recorded biochemical pregnancy will be negative if any of the following occur:

- Study is recorded as complete.
- No eggs are collected.
- No oocytes injected with sperm.

- Normal fertilisation not seen and no data recorded for embryo development.
- All embryos under development Frozen or Degenerated and destroyed.
- No embryos transferred.
- Study not completed and reason for not completing study indicates no biochemical pregnancy. Reasons for not completing the study will be reviewed by the chief investigator prior to unblinding of the treatment allocations.

If no results from biochemical pregnancy tests are recorded biochemical pregnancy will be missing if:

- None of the above criteria are met.

If a clinical pregnancy or positive fetus outcome is subsequently recorded biochemical pregnancy will be considered positive regardless of the results of the biochemical pregnancy test or absence of results from a biochemical pregnancy test.

Secondary Outcomes

Clinical pregnancy at 6-9 weeks gestation

Clinical pregnancy will be positive if any of the following occur:

- Clinical pregnancy is confirmed in USG (Variable 'Cpregn' = 1) with gestational age at least 6 weeks.
- The first USG for clinical pregnancy is "non-diagnostic" and clinical pregnancy is confirmed in second USG scan (Variable 'Cpregn' = 2 & 'Cpregn2' =1) with gestational age at least 6 weeks.
- Clinical pregnancy is confirmed in a USG scan with gestational age less than 6 weeks and at least one fetus has a recorded fetus outcome with gestational age at least 6 weeks.
- A live birth is subsequently recorded. In this instance clinical pregnancy will be considered positive regardless of the results recorded for any clinical pregnancy scans or the absence of clinical pregnancy scans.

Clinical pregnancy will be negative if any of the following occur:

- A negative diagnostic USG scan for clinical pregnancy is recorded.
- Biochemical pregnancy is negative and no live births are recorded.
- Biochemical pregnancy is positive, but no diagnostic clinical pregnancy scans are carried out and the participant is recorded as "completing the study".
- Positive clinical pregnancy scan carried out with gestational age less than 6 weeks and fetus outcomes indicating pregnancy end are recorded with gestational age less than 6 weeks for all fetuses. Fetus outcomes include miscarriage and termination.

Clinical pregnancy will be missing if any of the following occur:

- Biochemical pregnancy is missing Clinical pregnancy scan performed before week 6 and gestational age is missing for all fetus outcomes.
- Clinical pregnancy scan performed before week 6 and fetus outcomes are missing for all fetuses.
- no results from diagnostic USG scans for clinical pregnancy are recorded, participant is recorded as “not completing the study”, and reason given for not completing the study does not rule out clinical pregnancy. Reasons for not completing the study will be reviewed by the chief investigator prior to unblinding of the treatment allocations.

Live birth at < 37 weeks gestation

Live birth at < 37 weeks will be positive (and a negative or missing clinical pregnancy will redefined as positive) if:

- Fetus outcome is live birth and gestational age is less than 37 weeks for one or more fetuses

Live birth at < 37 will be negative if either of the following occur:

- Clinical pregnancy is negative
- Clinical pregnancy is positive and either the fetus outcome is not live birth, or gestational age is greater than or equal to 37 weeks, for all fetuses.

Live birth at < 37 will be missing if any of the following occur:

- Clinical pregnancy is positive or missing and there is no recorded fetus outcome data
- Gestational age is missing for all fetuses with fetus outcome of live birth

Miscarriage (defined as pregnancy loss after confirmation of clinical pregnancy)

Miscarriage will be positive if:

Pregnancy end is ‘miscarriage’ for any registered fetus (Variable ‘PregEnd’ = 1) and clinical pregnancy (as described above) is positive.

Miscarriage will be negative if either of the following occur:

- Clinical pregnancy is negative.
- Clinical pregnancy is positive and fetus outcome is not miscarriage for all fetuses.

Miscarriage will be missing if either of the following occur:

- Clinical pregnancy is positive or missing and there is no recorded fetus outcome data.

Primary Outcome

Live birth ≥ 37 weeks gestation

Live birth at ≥ 37 weeks gestation will be positive (and a negative or missing clinical pregnancy will redefined as positive) if:

- Pregnancy end reason is ‘Live Birth’ for any registered fetus (Variable ‘PregEnd’ = 6) and Gestational age for the corresponding baby is ≥ 37 weeks (Variable ‘NOGAge’ ≥ 37)

Live birth at ≥ 37 weeks gestation will be negative if either of the following occur:

- Clinical pregnancy is negative
- Fetus outcome is not live birth, or fetus outcome is live birth and gestational age is < 37 weeks, for all fetuses

Live birth at ≥ 37 weeks gestation will be missing if any of the following occur:

- Clinical pregnancy is positive or missing and there is no recorded fetus outcome data.
- Gestational age is missing for all fetuses with fetus outcome of live birth.
